# Unlocking the potential: analyzing 3D microstructure of small-scale cement samples from space using deep learning

**DOI:** 10.1038/s41526-024-00349-9

**Published:** 2024-01-25

**Authors:** Vishnu Saseendran, Namiko Yamamoto, Peter J. Collins, Aleksandra Radlińska, Sara Mueller, Enrique M. Jackson

**Affiliations:** 1 Department of Aerospace Engineering, The Pennsylvania State University, University Park, 16802 PA USA; 2 Department of Civil and Environmental Engineering, The Pennsylvania State University, University Park, 16802 PA USA; 3 Department of Ecosystem Science and Management, The Pennsylvania State University, University Park, 16802 PA USA; 4 NASA Marshall Space Flight Center, Huntsville, 35808 AL USA

**Keywords:** Aerospace engineering, Engineering

## Abstract

Due to the prohibitive cost of transporting raw materials into Space, in-situ materials along with cement-like binders are poised to be employed for extraterrestrial construction. A unique methodology for obtaining microstructural topology of cement samples hydrated in microgravity environment at the International Space Station (ISS) is presented here. Distinctive Scanning Electron Microscopy (SEM) micrographs of hardened tri-calcium silicate (C_3_S) samples were used as exemplars in a deep learning-based microstructure reconstruction framework. The proposed method aids in generation of an ensemble of microstructures that is inherently statistical in nature, by utilizing sparse experimental data such as the C_3_S samples hydrated in microgravity. The hydrated space-returned samples had exhibited higher porosity content (~70 %) with the portlandite phase assuming an elongated plate-like morphology. Qualitative assessment of the volumetric slices from the reconstructed volumes showcased similar visual characteristics to that of the target 2D exemplar. Detailed assessment of the reconstructed volumes was carried out using statistical descriptors, and was further compared against micro-CT virtual data. The reconstructed volumes captured the unique microstructural morphology of the hardened C_3_S samples of both space-returned and ground-based samples, and can be directly employed as Representative Volume Element (RVE) to characterize mechanical/transport properties.

## Introduction

With the advent of crewed missions as part of the Artemis program^1^, interests are being renewed to sustainably prolong human space expeditions. Invariably, this requires infrastructure on extraterrestrial bodies to protect both humans and equipment from the extreme environments. Given the cost of transporting materials for deep space missions, it is envisioned that in-situ resources such as lunar regolith formed into cement-like binders can be employed for constructing habitats on extraterrestrial bodies. However, there is very little understanding on the hydration of cement-like binders in space. A recent study on cement solidification in microgravity environment helped understand the chemistry of hydration and microstructural formation in the absence of gravity^2,3^. To gain deeper understanding on the impact of microstructural morphology on the resulting mechanical properties of cement, experimental or numerical simulations may be employed. Moreover, tricalcium silicate (C_3_S) [Ca_3_SiO_5_: C_3_S in cement notation], which constitutes ~50–70 % of Ordinary Portland Cement (OPC) by mass^4–9^, is an important component governing the hydration of OPC. Hence, the C_3_S microstructure directly influences the physical properties of hardened cement paste. Owing to the size limitations and high porosity of the space-returned hydrated C_3_S samples^2^, conventional experimental characterization techniques are not viable. Therefore, numerical modeling is currently the only way to evaluate mechanical properties and perform structure-property predictions of such a highly porous material. To estimate the mechanical and transport properties of cementitious systems using a numerical code, 3D representations that accurately capture the microstructure morphology are necessary. An obvious way is to use Microtomography (micro-CT), noticing its limitations – (1) it becomes costly when trying to evaluate statistical data of every sample, and (2) due to size limitation or poor material contrast, some samples cannot be measured. Note that 2D imaging is commonly used in cement and concrete science, but that does not allow for adequate representation of 3D features. A cost-effective method is to use microstructure reconstruction techniques that computationally generate statistically equivalent microstructure of a target 2D exemplar.

In this study, to obtain comparative mechanical properties, sub-volumes of C_3_S samples hydrated in both microgravity (μ*g*) and earth (1*g*) were generated using a deep learning-based texture synthesis architecture. The reconstructed volumes containing microstructural features may be directly employed as Representative Volume Elements (RVEs) in numerical codes. There is a high discrepancy in the observed microstructural morphology in 1*g* and μ*g* samples^2^. The deep learning-based reconstruction approach provides an efficient way to create a fairly large ensemble of microstructures to generate design allowables. The high-resolution 2D images obtained using SEM stored in the NASA PSI database were employed for 2D to 3D reconstruction.

In the domain of computational materials science, aptly representing microstructure is integral for the exploration of physical and mechanical properties of materials. Due to the high cost and effort involved in 3D material characterization, advanced reconstruction methodologies are preferred to generate statistically equivalent 3D volumes from high-fidelity 2D scans. The crux of microstructure reconstruction methodologies involves representing the material internal structure via statistical descriptors, as opposed to a deterministic description achieved in conventional 3D scans. To reliably establish process-microstructure-property linkage, it is vital to characterize material properties using a fairly large virtual dataset. Hence, the ensemble of microstructures that gives a comprehensive statistical representation of the material is preferred to predict homogenized macroscale properties^10^. In the literature, there exist several microstructure reconstruction methodologies, which may be broadly classified as statistical modeling-based, visual features-based and AI-based^11^.

Typically, statistical representation of microstructure from a given 2D exemplar is achieved using statistical functions^12–18^ and physical descriptors^19–24^. The former employs a stochastic optimization strategy such that the chosen statistical features (such as probability, lineal-path and cluster functions^25^) of the exemplar closely match the reconstructed 3D microstructure. In the latter approach, physical descriptors of the exemplar (e.g, grain and pore size) are matched with that of the reconstructed microstructural volume. With the advent of AI-based models in the field of computer vision, several deep learning approaches based on Convolutional Neural Networks (CNNs) are widely becoming popular as they are well-suited to handle image data^26–29^.

In the context of microstructure reconstruction, deep learning approaches may be classified as^11^: material-system-dependent and material-system-independent. Notable works on the material-system-dependent deep learning approaches include the ones by Cang et al.^30^ and Li et al.^31^. These methods train the weights of the employed network with images specific to a particular material and needs to be retrained for a new material. On the other hand, transfer learning approaches are material-system-independent and circumvents the need for training weights with a set of materials data. Such deep learning models employ pre-trained weights using benchmarked datasets from the field of computer vision. A few notable works based on transfer learning approaches include the ones by Lubbers et al.^28^, Li et al.^11^, and Bostanabad^26^. These models adopted the deep learning architecture, VGG-19^32^, trained on ImageNet database^33^, and used the activations of its network layers to generate reconstructions for a given target microstructure. Note that such models still may require hyperparameter tuning to get the most optimal results for a given material system.

By characterizing the exemplar as a Markov Random Field (MRF), reconstruction methods based on texture synthesis^34–38^ are widely becoming popular over the optimization-based approaches. By defining the 2D exemplar as a Markovian field, Lubbers et al.^28^ utilized activations of deep convolutional layers developed by Gatys et al.^39^ to synthesize microstructures with same texture representation. However, note that statistical equivalency cannot be achieved in microstructure reconstruction by visual similarity alone. The texture synthesis method is efficient as it achieves reconstruction in a single pass, in contrast to global and iterative procedures used in the traditional optimization approach^35^. In addition, this approach preserves the material descriptors (e.g., grain shape), and enables extending material modeling to anisotropic materials and can synthesize 3D microstructure from 2D cross-sections.

Recently, the deep learning-based reconstruction technique, a subset of machine learning-based approach has also received wide attention from the materials community^11,26,27,30^. In the field of computer vision and graphics, Solid Texture Synthesis (STS) methods^40,41^ are widely utilized to generate solid textures across a set of slicing directions for a given 2D exemplar^42–45^. The STS methods have been successfully applied to model 3D microstructure of various natural materials^10,35,46,47^, especially porous media^48,49^. For a given set of coordinates in the 3D space, using the STS method, a texture is added to a 3D surface by directly evaluating a colormap function. For instance, Kopf et al.^43^ synthesized 3D texture solids from 2D exemplars using a non-parametric texture optimization approach coupled with histogram matching. These STS methods are fully automated and are able to generate a broad set of textures. In short, the method addressed the ill-posed nature of the problem by assuming that the exemplar is a stationary texture and has spatial locality property. More recently, Gutierrez and co-workers^50^ proposed another framework of STS which adopts a compact solid texture generator model that takes a multi-scale noise input and produces a 3D solid texture based on a perceptual slice-based loss function. To characterize the synthesized textures and optimize the loss function, the activations in the hidden layers of pre-trained deep CNN, VGG-19^32^ was used. This proposed deep learning-based approach is computationally efficient and can be directly employed for synthesizing 3D cement microstructure from a high-resolution 2D exemplar^51,52^.

The goals of this work are 1) to develop an AI-assisted framework to quantitatively characterize the 3D microstructures of space-returned cement samples using statistical functions, and 2) to effectively reconstruct the 3D models based on 2D exemplars through sensitivity study, aimed at samples with high porosity ( > 45 %) and of anisotropic-shaped phases. The space-originated samples, including C_3_S cement samples studied in this work, are expected to have higher porosity and unique phase growth due to the effect of microgravity. Two impacts are expected from this work: 1) quantitative information about 3D microstructure information can be obtained even when only 2D images are available about samples that are limited in size and quantity, and 2) the validated reconstructed 3D models can be useful for process-structure-relationship study, to be compared with results from hydration process modeling and to be used as an input for micromechanics-based modeling. The generative framework applied to volumetric texture synthesis in the field of computer vision^50^ was used here to generate high-quality virtual volumes of cement samples hydrated in space and ground. This unique reconstruction methodology aided us in creation of statistical ensemble of microstructures, and facilitated description of the average 3D microstructure characteristics and their distribution in the space-returned samples. We validated the reconstructed volumes using statistical descriptors and further compared them against micro-CT virtual data. Subsequently, this led to comparison against the microstructural morphology of ground-based samples. The framework outlined here, that involves the generation of statistically equivalent virtual volumes and their subsequent validation, can be applied to advance materials research studies in space.

## Methods

### Process parameters - C_3_S hydration in microgravity and ground

The mixture comprised C_3_S mixed with lime-water at a water-to-cement (w/c) ratio of 2.0 by mass (5g of C_3_S and 10g of lime-water). Lime-water was used instead of pure water to circumvent the unrealistic rapid initial reaction at such high w/c. The experiments were conducted using commercially available plastic bags onboard the ISS as well on the ground. The plastic bags allow the water and cement to remain separated until the desired mixing time. Both space and ground samples were mixed at same condition: temperature 20 ± 2 °C, 1 ATM pressure and 35% relative humidity. The hydrating C_3_S paste was manually mixed until homogeneity is achieved. Both ground and space processed samples were allowed to remain in the sealed bag for the entire duration of hydration undisturbed held at controlled temperature 20 ± 2 °C. The space samples were allowed to hydrate aboard the ISS for 42 days prior to returning to Earth which allowed for a significant degree of hydration. Note, the only process variable between the space and ground is gravity. For more details on experimental setup and procedure refer to^2^.

### Microscope observations

Space samples returned to ground were immediately retrieved to conduct analysis and for subsequent comparison with the ground-based samples. Polished samples were prepared at day 152 after initial hydration, and Backscattered Electron (BSE) micrographs of fracture surfaces were obtained using FEI Q250 at 500x magnification^2^. In total, 20 images of space samples and 30 images of the ground samples were examined and stored in the NASA PSI database. The resolution of the BSE images [~1536 x 1024 pixels] was 0.54 *μm*. Exemplars for 3D reconstruction were then chosen from the NASA PSI database. Image analysis was carried out to discern the C_3_S hydration products - calcium silicate hydrate (C-S-H), portlandite (CH), and porosity in both space-returned and ground-based samples (C-S-H and CH; cement chemistry notation C = CaO, S = SiO_2_, and H = H_2_O followed throughout this paper).

### Micro-CT imaging and analysis

Micro-CT images were acquired using a Zeiss Xradia 620 Versa X-ray Microscope at the Penn State Center for Quantitative Imaging. For 1*g* and and μ*g* samples, a specimen size ~10 x 6 x 6 mm was extracted and fixed atop a specimen mount (Supplementary Fig. 1). For details on scan setup and parameters, see Supplementary Note 1 and Supplementary Table 1. The threshold bound to discern each phase was determined from the porosity, CH and C-S-H estimates resulting from the image analysis of BSE micrographs presented in Table 1. The phase-discerned 3D virtual samples (Supplementary Fig. 2) were then evaluated using the low-order probability distribution functions (see Supplementary Note 2).

**Table 1:** Summary of individual phase composition (%) of hydration products and porosity per image analysis (±SD) of 1*g* and μ*g* samples

	Ground (1*g*)	Microgravity (μ*g*)
Porosity	47.0 ± 14.2	70.3 ± 1.4
Porosity - MIP [2]	48.4	69.4
C-S-H	41.6 ± 12.0	21.2 ± 1.2
CH	11.3 ± 2.8	8.5 ± 1.4

### Deep learning-based microstructure reconstruction

**Fig. 1:**
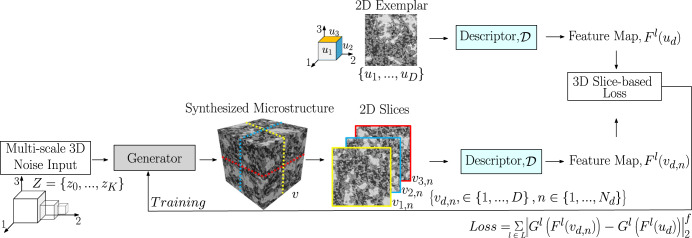
Deep learning-based 3D microstructure reconstruction of a target BSE image illustrated on 1*g* sample, where the CNN generator, ${{{\mathcal{G}}}}(Z| \theta )$, synthesizes the microstructural volume, **v**, from a 3D multiscale noise input, *Z*. 2D slices of the generated microstructure volumes, *v*_*d*,*n*_, are compared to the exemplar, *u*_*d*_, corresponding to desired view along the direction, $D\in \left\{1,2,3\right\}$ with the aid of perceptual slice-based loss function, ${{{\mathcal{L}}}}$, defined in Equation (1). Descriptor network, ${{{\mathcal{D}}}}$, based on pre-trained deep CNN, namely, VGG-19 is utilized here. Figure adapted with permission from^50^, John Wiley & Sons.

The details of the deep learning-based 3D reconstruction framework as originally described by Gutierrez et al.^50^ is briefly presented here. Figure 1 shows the training framework of the proposed generator network that produces a solid texture from a multi-scale 3D noise input, following the solid texture synthesis model^50^. This CNN-based model generates high-quality 3D virtual volumes that are statistically equivalent to the target exemplar(s). The training framework is illustrated on the 1*g* sample in Fig. 1, in which a convolutional neural generative network, ${{{\mathcal{G}}}}$, is trained to synthesize solid microstructure texture, ${\bf{v}}={\mathcal{G}}(Z|\theta )$, from a multi-channel 3D white noise inputs, *Z* = {*z*_0_, . . . , *z*_*K*_}. The CNN-based compact solid texture generator through multi-scale architecture of convolution, concatenation and upsampling operations transform the white noise, *Z*, into a solid texture, **v**. It starts at the coarsest scale, wherein, the volumetric noise sample is processed with a set of convolutions, followed by an upsampling operation to reach the next scale. This is then concatenated with an independent noise sample from the next scale that is also processed with a set of convolutions. This proposed fully convoluted neural generator network allows for synthesizing rectangular volume textures of any arbitrary size driven by the size of the input. For more details about these blocks of operation used in the generator network following a multi-scale architecture, see^50^. The learnable generator parameters, *θ*, to produce the solid color texture, ${\bf{v}}={\mathcal{G}}(Z| \theta )$, are the weight, bias, mean and variance of the batch normalization layers in channel concatenation operation, and kernels and bias of the convolution block.

The generated volumes, ***v***, are then compared to the given exemplar(s) {*u*_1_, . . . , *u*_*D*_} corresponding to the desired view along $D\in \left\{1,2,3\right\}$ directions among the three-dimensional Cartesian coordinates. By optimizing the parameters, *θ*, the generator reproduces solid textures using the features extracted from the 2D exemplar. This is achieved by a 3D slice-based loss function, by comparing the feature map, **F**, of the 2D slices *v*_*d*,*n*_ (*n*^*th*^ slice along *d*^*th*^ direction) to that of the exemplar, *u*_*d*_. In a classical CNN-based optimization approach, batches of virtual volumes need to be synthesized during training which a priori requires a prohibitive amount of memory. To cope with such memory limitations and computational complexity inherent to high resolution exemplars (for instance, pixel resolution of BSE micrograph utilized as exemplar in Fig. 1 is 0.54 *μm*), only comparisons between 2D slices, *v*_*d*,*n*_, of the synthetic solid and exemplar, *u*_*d*_, are performed during training. Here, the feature maps of both target image and 2D slices of the generated solid microstructure are extracted using the pre-trained deep CNN descriptor (${{{\mathcal{D}}}}$) network, VGG-19^32^. The feature maps, **F**^**l**^, are intermediate outputs in a pre-trained deep CNN. When the descriptor, ${{{\mathcal{D}}}}$, is evaluated on an image, it results in feature maps, ${{{\mathcal{D}}}}:\in {{\mathbb{R}}}^{{N}_{1}\times {N}_{2}\times 3}\mapsto {\{{{{{\bf{F}}}}}^{{{{\bf{l}}}}}({{{\bf{x}}}})\in {{\mathbb{R}}}^{{N}^{l}\times {M}^{l}}\}}_{l\in L}$, where *L* is the VGG-19 layers considered, with each layer, *l*, comprising *N*^*l*^ spatial values and *M*^*l*^ channels. Gram matrix, ${{{{\bf{G}}}}}_{{{{\bf{i}}}},{{{\bf{k}}}}}^{{{{\bf{l}}}}}={\sum }_{k}{{{{\bf{F}}}}}_{{{{\bf{i}}}},{{{\bf{k}}}}}^{{{{\bf{l}}}}}{{{{\bf{F}}}}}_{{{{\bf{j}}}},{{{\bf{k}}}}}^{{{{\bf{l}}}}}$, aids in understanding how similar the feature map, ${{{{\bf{F}}}}}_{{{{\bf{i}}}},{{{\bf{k}}}}}^{{{{\bf{l}}}}}$ is to its transpose, ${{{{\bf{F}}}}}_{{{{\bf{j}}}},{{{\bf{k}}}}}^{{{{\bf{l}}}}}$; where *l* = number of layers, *k* = number of channels. The dot product (${{{{\bf{G}}}}}_{{{{\bf{i}}}},{{{\bf{k}}}}}^{{{{\bf{l}}}}}$) gets larger when the feature vectors get more similar. Thus, the Gram matrix, **G**, characterizes how well correlated are the textures using the feature maps represented as VGG statistical features of the microstructure. A Gram matrix-based loss function is then defined, which is a measure of the difference in textures of the target image and the 2D slices of the reconstructed microstructure. On training, the parameters of the generator, *θ*, are optimized by minimizing the loss function between the exemplar, *u*_*d*_, and 2D slices, *v*_*d*,*n*_, extracted from the synthesized volume^50^:

1 $$Loss=\sum\limits_{l\in L}\left\|{\bf{G}}^{\bf{l}}\left({\bf{F}}^{\bf{l}}({\bf{v}}_{{\bf{d}},{\bf{n}}})\right)-{\bf{G}}^{\bf{l}}\left({\bf{F}}^{\bf{l}}({\bf{u}}_{{\bf{d}}})\right)\right\|_{2}^{f}$$

where ${\left\Vert \cdot \right\Vert }_{f}$ is the Frobenius norm, *v*_*d*,*n*_ denotes the slices of the generated 3D microstructure, and *u*_*d*_ is the BSE exemplar. The loss function (Eq. (1)) is evaluated on 2D slices extracted from the synthesized microstructure (Fig. 1), leading to a memory efficient training. In the reconstructed 3D microstructure, ${{{\bf{v}}}}\in {{\mathbb{R}}}^{t\times h\times w\times 3}$ (4D tensor of size - thickness, height, width and three-channel, i.e., RGB), ${v}_{D}^{n}$ is given as the *n*^*th*^ 2D slice of the generated solid orthogonal to the *d*^*th*^ direction. This efficient single-slice based training enables utilization of high resolution 2D micrographs as exemplars. The current reconstruction framework can also be easily adapted to input grayscale images (single-channel) without any significant modification^52^, and was thus utilized with μ*g* and 1*g* BSE micrographs. The parameters were optimized using the Adam algorithm^53^ with an initial learning rate 0.1 over 3000 iterations. For all reconstructions, a multi-step learning rate scheduler was employed which decayed the learning rate by a factor 10 at epochs 300, 1000 and 2000.

### Reporting summary

Further information on research design is available in the Nature Research Reporting Summary linked to this article.

## Results and Discussion

### Image analysis of 2D exemplars

The first objective was to discern the C_3_S hydration products - calcium silicate hydrate (C-S-H), portlandite (CH), and porosity in both space-returned and ground-based samples. The raw BSE images stored in the NASA PSI database were used. To identify the bounds between the various phases, a greyscale histogram-based image segmentation utilizing the overflow method^54^ coupled with the local minima was employed. The overflow method has been proven to identify the histogram bound between phases that have a large difference between their atomic numbers, for instance, porosity and C-S-H matrix. The boundary between C-S-H and CH, may then be identified from a local minimum value. In addition, prior to the segmentation, the BSE images were subjected to histogram enhancement (with 0.3% saturated pixels) followed by Sigma filter^55^ (*σ* = 2.0). For image analysis in cementitious systems, the Sigma filter has been widely employed, especially to identify the Interfacial Transition Zone (ITZ) between the cement paste and aggregates in concrete^56^. The workflow of greyscale histogram-based BSE image segmentation followed by phase identification is provided in Supplementary Fig. 4.

**Fig. 2:**
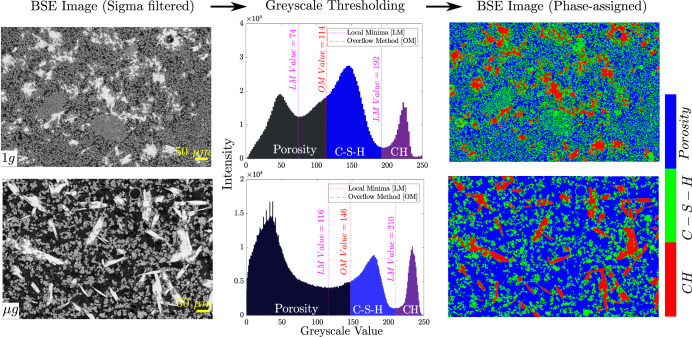
Raw BSE micrographs modified by histogram enhancement followed by sigma-filter (left column). Greyscale histogram-based thresholding utilizing the overflow method was employed to identify the porosity bounds (middle column). Phase assigned BSE images of hydrated C_3_S 1*g* and μ*g* samples (right column) [size: ~1536 x 1024 pixels, magnification: 500x].

The phase-segmented BSE images were then used to compute area fraction of each phase. The porosity content in the samples were evaluated using Mercury Intrusion Porosimetry (MIP)^2^. The area fraction of porosity (averaged across all BSE images) in both 1*g* and μ*g* samples were found to be in good agreement with the results obtained from MIP (Table 1). It should be noted that the porosity presented here is the total porosity, the value measured after mercury infiltrated throughout the sample. MIP exhibits its limitation with identifying pore size distribution, however, this total porosity measurement is useful for comparative study in our work. The identified greyscale bounds were used to visualize various phases in the samples and are presented in Fig. 2. Both CH and C-S-H phase fractions have not been evaluated in the past, and were obtained here for both 1*g* and μ*g* samples, see Table 1.

In addition to the high porosity in μ*g* samples (70%; as opposed to 47% in 1*g* samples), a stark difference in size, shape and distribution of the portlandite phase was also observed (Fig. 2). In the case of 1*g* samples, the CH crystals are uniformly distributed in the C-S-H matrix. Whereas, in μg samples, due to the lack of directional force they assumed an elongated plate-like morphology. The spatial pore size distribution directly affects the physical and mechanical properties of the hydrated cement samples. Therefore, from the perspective of exploring process-structure-property relationship, it is meaningful to have a statistical representation of the microstructure as opposed to a deterministic one, obtained by interrogating a certain instantiation of small subvolumes.

### Qualitative assessment of microstructure reconstruction

**Fig. 3:**
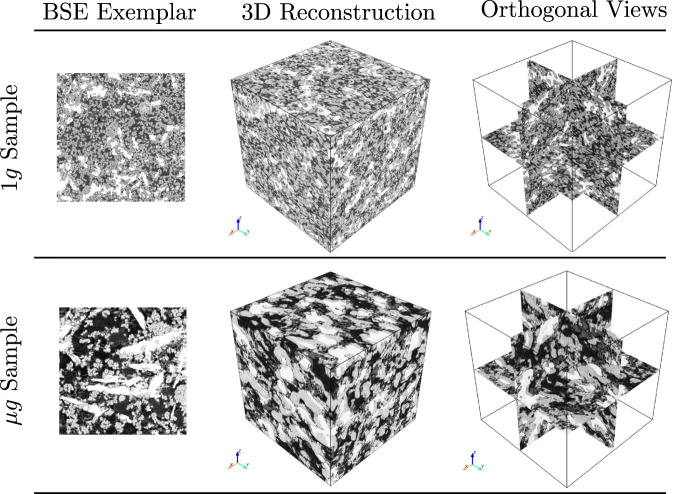
Qualitative comparison of reconstructed 3D microstructure and their respective orthogonal views along with the 2D exemplars for both 1*g* and μ*g* samples. Chosen BSE exemplar size: 512 x 512 pixels (resolution 0.54 μm, edge-length 276.5 μm).

**Fig. 4:**
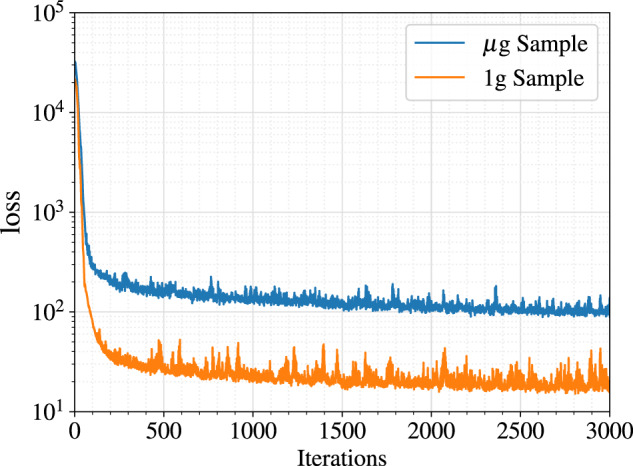
Loss function plotted against number of iterations for 1*g* and μ*g* samples. BSE exemplar size - 512 x 512 pixels, resolution 0.54 μm.

The primary objective of this analysis was to compare the visual characteristics of the reconstructed microstructures with the chosen 2D exemplars. The reconstructed 3D microstructures of both 1*g* and μ*g* hydrated samples, and the respective BSE exemplar are provided in Fig. 3. An initial exemplar size 512 x 512 pixels (276.5 x 276.5 μm^2^) was chosen randomly from an original micrograph of size 1536 x 1024 pixels. The orthogonal sections show that the microstructural topology of the exemplars are very well captured in both reconstructed 1*g* and μ*g* samples. In particular, the elongated plate-like characteristics of the CH crystal is visible in the orthogonal slices (see Fig. 3). The deep learning-based model employed here extracts the microstructural features using a fixed descriptor network, VGG-19^32^, and synthesizes a volume that is statistically equivalent to the target image. In the case of μg samples, the challenge is to reproduce the rather elongated plate-like morphology of the portlandite phase, as well as the spatial distribution of porosity. The value of the loss function during training of 1*g* and μ*g* samples is provided in Fig. 4. Convergence occurred after 1500 iterations for both samples for an exemplar size of 512 x 512 pixels.

**Fig. 5:**
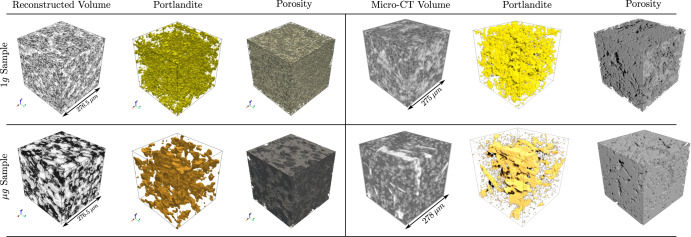
Qualitative comparison of reconstructed 3D microstructure against micro-CT virtual volume for 1*g* and μ*g* hydrated samples. The portlandite phase and porosity for each sub-volume was extracted using greyscale histogram-based thresholding approach. A micro-CT virtual sub-volume with edge length ~275 μm was extracted to perform further quantitative evaluation.

Furthermore, micro-CT virtual samples were also compared against the reconstructed microstructures of both 1*g* and μ*g* samples. Visual comparison with phase-segmented volumes (portlandite and porosity) following the greyscale histogram-based segmentation is provided in Fig. 5. Micro-CT sub-volume was extracted to compare against the reconstructed 3D volumes using 512 x 512 pixels exemplars (resolution 0.54 *μm*). The distinct plate-like morphology of the μ*g* sample was visible in both the reconstructed and micro-CT virtual sample (Fig. 5). In the case of 1*g* sample, the portlandite phase is uniformly distributed, and was well captured in the reconstructed volume. The micro-CT virtual images further corroborated this observation. The synthesized 3D volumes of both samples showed similar microstructural characteristics to that of the 2D target images, as well as micro-CT virtual data, showcasing that the methodology can generate realistic ensemble of 3D microstructures.

### Microstructure reconstruction validation using statistical descriptors

The hydrated cement paste (both 1*g* and μ*g* samples) contain random pore distribution, e.g., see the segmented porosity phase of 1*g* sample in Fig. 5. Moreover, the CH and C-S-H phases also follow clustering and connectivity such that one of the characterization approaches is probabilistic representation. Among such class of methods, the low-order probability distribution functions - two-point correlation function, *S*_2_(*r*), lineal-path function, *L*_2_(*r*), and two-point cluster function, *C*_2_(*r*) have been found to be effective^14,25^. In this work, these low-order probability functions have been adopted as quantitative evaluation metrics to compare the statistical equivalence of microstructure reconstruction to that of the micro-CT virtual sample (ground truth). These functions were used for statistical and quantitative description of a particular phase under consideration (see Supplementary Note 2 for details). Note, owing to the uncertainty surrounding the randomness of individual phase distribution, the mechanical properties (stiffness and strength) can exhibit fluctuations among the reconstructed microstructural volumes. The two-point correlation function, *S*_2_(*r*), describes short-range phase connectivity, whilst the two-point cluster function, *C*_2_(*r*), aids in describing specific characteristics of the spatial distribution and continuity of clustering, such as percolation of a particular phase. The lineal-path function, *L*_2_(*r*), characterizes long-range phase connectivity. Furthermore, these low-order statistical functions were employed to characterize voids in concrete and correlate them to both mechanical and physical properties, such as hydraulic conductivity^57,58^. Here, the spatial distribution of portlandite and porosity content within the synthesized 3D volume is compared against micro-CT virtual samples using the statistical descriptors as a means of quantitative validation. Moreover, these low-order functions also serve as indicators for the applicability of the chosen volume to serve as a RVE^52^.

**Fig. 6:**
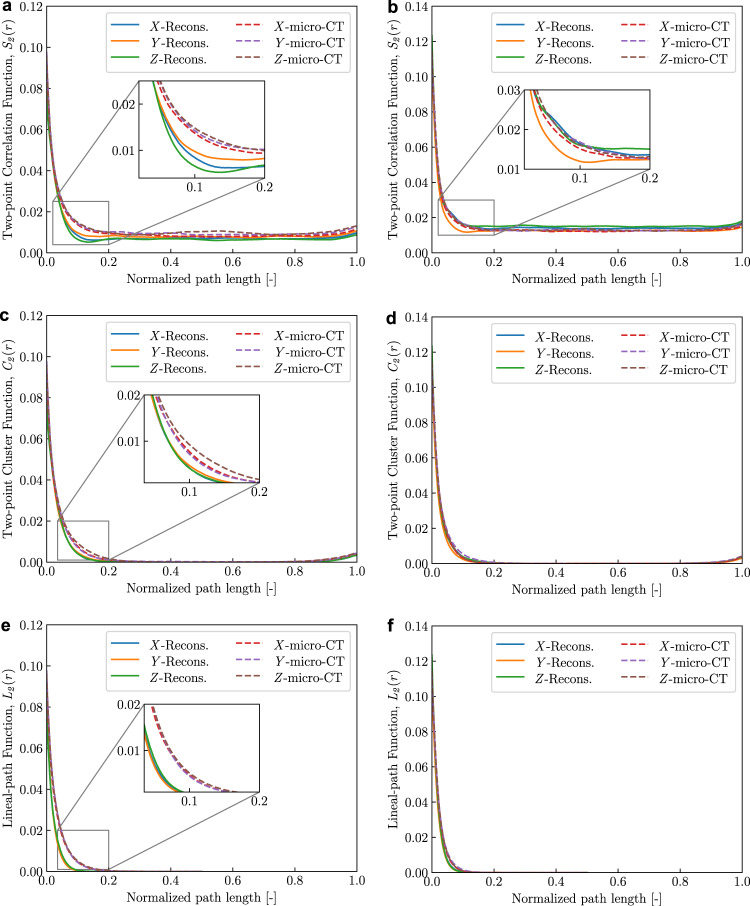
Quantitative evaluation of reconstructed topology of the portlandite phase using statistical descriptors: two-point correlation function, *S*_2_(r). (**a**) μ*g* sample, (**b**) 1*g* sample; two-point cluster function, *C*_2_(r) (**c**) μ*g* sample, (**d**) 1*g* sample and lineal-path function, *L*_2_(r) (**e**) μ*g* sample, (**f**) 1*g* sample. Volume edge-length ~ 275 μm; 2D exemplars (512 x 512 pixels) from Fig. 3.

Characterizing the spatial distribution of portlandite clusters in both 1*g* and μ*g* samples is vital, as the connectivity and distribution of the portlandite phase strongly governs the mechanical and transport properties in hydrated cement pastes. Hence, the statistical descriptors can be used to fully describe the morphology of the portlandite phase as well as porosity by extracting them from both reconstructed and micro-CT sub-volumes (Fig. 5). Figure 6 presents the low-order probability functions - *S*_2_(*r*), *C*_2_(*r*), and *L*_2_(*r*) with mean fit evaluated against the portlandite phase for both 1*g* and μ*g* samples. The functions were evaluated on 2D slices of three orthogonal cut plane sweeps for both micro-CT and reconstructed sub-volumes. The distance between two points, *r* of a test vector is normalized with the edge length of the considered volume. For all volumes, probability functions are obtained in *X*, *Y*, and *Z* direction.

A high *S*_2_(*r*) indicates that more phase clusters exist in that specific direction^16,17^. In the reconstructed 1*g* volume, the CH phase is clustered along *X* and *Y* directions between normalized path length 0.1 and 0.2 (Fig. 6b). On the other hand, micro-CT virtual sample exhibited only very little variation within the same limits along all three directions. In addition, the reconstructed volumes captured both phase connectivity and isotropy of the portlandite phase in the case of 1*g* sample, represented by plots in Fig. 6d and f, respectively. The *S*_2_(*r*) values of the reconstructed μ*g* sample volume showed minor perturbations between ~0.08 and 0.2 due to the difference in the initial slope (Fig. 6a). This indicates that relatively more clustered portlandite content is present in the micro-CT virtual sample compared to the reconstructed volume.

The two-point cluster function *C*_2_(*r*) provide information about the connectivity and clusters of a particular phase and can be used to study the spatial distribution of portlandite such as clustering and connectivity in both micro-CT and reconstructed volumes. In case of μ*g* sample, it can be noted from the micro-CT virtual sample that *C*_2_(*r*) is slightly higher in the *Z* direction, indicating that portlandite clusters dispersed along the *Z* direction is larger than the other directions (Fig. 6c). This is indicative of the previously mentioned plate-like morphology of the CH phase in the μ*g* sample. In general, the reconstructed microstructural volume presented here, captured the portlandite cluster dispersion observed in the μ*g* sample. However, between 0.1 and 0.2 normalized path length, it is found that *C*_2_(*r*) varies slightly between the reconstructed and micro-CT volumes. In addition, the anisotropy of the portlandite distribution for both reconstructed and micro-CT volumes can be noted in Fig. 6c. Plots in Fig. 6d and d indicate that *C*_2_(*r*) converges to zero indicating discontinuous portlandite cluster formation. This is also evident from the visual inspection of both reconstructed and micro-CT volumes in Fig. 5.

*L*_2_(*r*) values for both reconstructed and micro-CT volumes along *X*, *Y* and *Z* directions are same, indicating isotropic phase connectivity of the portlandite (Fig. 6e). The deep learning-based methodology successfully captured the portlandite connectivity in the 1*g* sample as noted in Fig. 6f. The *L*_2_(*r*) values for both reconstructed and micro-CT volumes are identical in this case. It can also be noted that *L*_2_(*r*) is zero when normalized path length is over ~ 0.2 for both samples, indicating that no portlandite clusters pass parallel to *X*, *Y*, and *Z* directions.

**Fig. 7:**
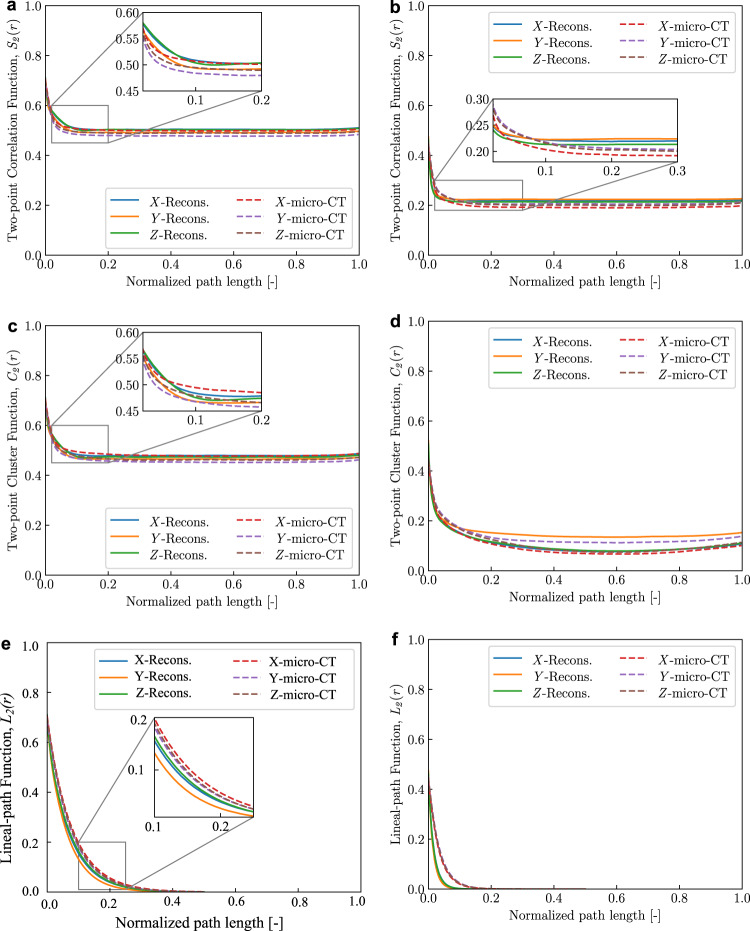
Quantitative evaluation of porosity content using statistical descriptors: two-point correlation function, *S*_2_(r). (**a**) μ*g* sample, (**b**) 1*g* sample; two-point cluster function, *C*_2_(r) (**c)** μ*g* sample, (**d**) 1*g* sample and lineal-path function, *L*_2_(r) (**e**) μ*g* sample, (**f)** 1*g* sample. Volume edge-length ~ 275 μm; 2D exemplars (512 x 512 pixels) from Fig. 3.

The mean fit of probability functions for the porosity content is provided for both 1*g* and μ*g* samples (Fig. 7). Unlike the portlandite phase, porosity connectivity is not restricted to any specific direction (for instance, see Fig. 5). Minor variation in inflection point ( ~ at 0.15) of the *S*_2_(*r*) values was observed between the micro-CT and reconstructed volumes for 1*g* sample [Fig. 7b]. The two-point cluster function, *C*_2_(*r*) is used to represent the spatial distribution of porosity such as clustering and connectivity in both micro-CT and reconstructed volumes. It is observed that *L*_2_(*r*) approaches zero when the normalized path length is over 0.5 (Fig. 7e and f), as there are no porosity clusters that pass through the volumes parallel to all three directions. However, as noted in Fig. 7c and d, *C*_2_(*r*) does not converge to zero indicating that continuously connected porosity clusters do exist in both samples that pass through the whole volume. This can also be visually confirmed from the extracted porosity phase (Fig. 5). Here, for both samples the spatial distribution of porosity in terms of clustering and connectivity was well represented in the reconstructed volume.

The quantitative analysis carried out on both portlandite and porosity phases using the low-order statistical functions helped to evaluate the volumes synthesized using the deep learning-based approach. As noted from the plots in Figs. 6 and 7, the deep learning-based model is able to aptly represent the 1*g* sample. In case of μ*g* sample, despite the minor perturbations, this approach can be utilized to generate a statistical ensemble leading to a probability analysis, rather than a deterministic one. Although the synthesized microstructures differ from the measured virtual samples visually, their variation using probability distribution functions are minor. Furthermore, this also implies that the statistical descriptors only capture lower-order statistical equivalence here. Hence, high-order evaluation metrics, such as moment invariants^38^ may be utilized and will be the subject of our future work. It must be noted that the reconstruction methodology currently employs the pre-trained network VGG-19. Ideally, in the domain of 2D to 3D microstructure reconstruction involving transfer learning approaches, a materials science-philic descriptor network such as MicroNet^59^ is preferred. Study on the influence of optimum descriptor network for microstructure reconstruction will considered in our future work. Currently, there does not exist any computational method to obtain micro-scale geometric model of cement hydrated in space (except micro-CT scanning). Hence, the presented methodology fills this gap by generating RVEs to investigate process-structure-property linkage of such space-cured material systems. Additionally, the presented methodology can also be employed for the generation of microstructures of hydrated cement paste (ground-based), and unlike the state-of-the art, circumvents the need to know the parameters such as mineral composition, water/cement ratio, cement fineness, particle size distribution as a priori^60–64^.

### Implications of exemplar size & resolution on 3D reconstruction

The objective of this analysis was to understand the field of view and resolution of the 2D exemplar, and their influence on 3D microstructure reconstruction using the proposed deep learning-based methodology. For accurate representation of various hydration phases in the reconstructed volumes, especially in case of μ*g* samples, wherein, elongated plate-like portlandite phase occurs, selection of 2D exemplar is vital. The field of view of the 2D exemplar must be such that the ~100 − 115 μm long portlandite phase is aptly captured. Hence, with a spatial resolution of 0.54 μm, at least ~200 x 200 pixels are required to amply capture the portlandite phase in the μ*g* sample. During training, the extracted feature maps of the 2D exemplar is compared against a perceptual loss function. Thus, the exemplar that is inputted in the solid texture synthesis framework (Fig. 1) governs the characteristics of the synthesized microstructure. The statistical features of the exemplar represented as feature map is acquired by the descriptor. Hence, the chosen exemplar must contain relevant phases for efficient microstructural reconstruction. Therefore, a few guidelines in relation to the selection of exemplar for training pertaining to the μ*g* sample is provided here based on a sensitivity study (see Supplementary Discussion for details).

To minimize utilization of computational resources and thus reduce training time, high fidelity smaller exemplar size is preferred (Supplementary Fig. 5). Training a network utilizing an exemplar of size 512 x 512 pixels and VGG-19 would require more than 12 GB memory^50,52^. As GPU memory is a limiting factor during the training process, it was decided to choose ROI of size 256^2^ and 512^2^ pixels as exemplars. The microstructural topology represented in each ROI differs (Supplementary Fig. 6).

Based on common design guidelines, the recommended RVE size for mechanical characterization must be at least 5 – 10x that of the characteristic heterogeneity size of the phase. For hydrated cement paste, typically RVE size in the range 150 – 200 μm is recommended^65,66^. To highlight the effect of exemplar size on reconstruction of 3D microstructures of both samples, statistical descriptors were utilized to aid the sensitivity study (Supplementary Fig. 7). The portlandite phase in 1*g* sample is uniformly distributed in the matrix (Fig. 2). Owing to the high resolution of BSE micrographs (0.54 μm / pixel) here, an exemplar of size 256 x 256 pixels (138.2 x 138.2 μm^2^) is sufficient for 1*g* sample. For the given resolution, a 512 x 512 pixels (276.5 x 276.5 μm^2^) exemplar size is recommended for the μ*g* sample.

An advantage of the deep learning-based model presented here or any similar model, is that the synthesized volume is not constrained to size of the 2D exemplar. In the absence of a high resolution or input image that is limited by size, the presented methodology can be used to generate microstructural volumes that are larger than that of the exemplar. This also paves the way in synthesizing not just 3D micro-scale models of hydrated cement paste as demonstrated here, but also 3D macroscopic mortar and concrete models augmenting to the current state-of-the art^67–69^.

A prime challenge of texture synthesis algorithms (or in general, for any reconstruction algorithm), is to provide a solution to the resolution vs. field of view dilemma. Here, the selection of exemplar window size and how it influences the reconstructed virtual volumes was demonstrated. Hence, in the absence of guidelines, a judicious selection of exemplar window with ample spatial resolution is recommended, especially when the reconstructed volumes are used to predict specific properties. This will circumvent simulations having spurious effects introduced by the artefacts in the reconstructed virtual volumes. Although, the current deep learning-based generative framework exhibits computational efficiency, one notable caveat concerning the reconstruction is when utilizing small exemplars. This is attributed to the descriptor network that are usually pre-trained using larger images (for instance, VGG-19 is trained on ImageNet dataset^33^). In addition, the most salient checker-board effects that are typical to generative texture-based stochastic reconstruction exists here too. One way to remove this artifact is by adding a weighted term to the loss function (Equation (1)). This weight combination will then need to be determined via a trial and error approach^27^. Moreover, influence of pre-trained descriptor networks, in particular, non-VGG architectures on microstructure reconstruction is also undetermined and is beyond the scope of current work.

In the field of materials science, data-driven modeling relying on 2D to 3D reconstruction has become a norm over the past few years to predict mechanical and/or physical properties. Here, a deep learning-based microstructure reconstruction approach was employed to investigate the microstructural characteristics of highly porous cement samples cured in a microgravity environment. The unique cement samples hydrated in space were limited by sample size and exhibited distinct microstructure morphology owing to the lack of gravity. Both qualitative and quantitative assessments indicated that the synthesized 3D microstructures are stable in comparison to micro-CT virtual data. In particular, reconstructed volumes successfully captured randomly oriented elongated plate-like morphology of the portlandite phase contained in space-cured samples. Moreover, the reconstructed volumes exhibited similar probabilistic distribution and were able to capture microstructural differences inherent to both space and ground. Random distribution of hydrated portlandite and porosity phases contained within a cement paste hydrated in space were characterized using low-order probability distribution functions. Through experimentation, the required minimum edge length of reconstructed virtual volumes to account for unique plate-like morphology (for a given exemplar resolution) of portlandite phase has been introduced. The reconstructed volumes presented here can be directly utilized as RVEs in advance micromechanical-based numerical codes for mechanical characterization. In addition, the presented work is computationally efficient, material-system independent, and can be easily extended to other multiphase materials and soil samples with sparse experimental data. Hence, the methodology presented here paves the way in creation of an ensemble of microstructures that are inherently statistical in nature (as opposed to fully deterministic) such as the space-hydrated cement, provided there is enough resolution in the exemplar to represent the various hydration phases. Thus, by generating statistical equivalent RVEs, probabilistic estimation of mechanical properties can be obtained using sparse experimental data and may be utilized as a standardized tool for upcoming materials research studies in space. In future, focus will be laid on incorporating higher-order evaluation metrics as well as validation of other material systems, such as typical heterogeneous materials.

## Supplementary information

Supplementary Information

Reporting Summary

## Data Availability

The 2D datasets that support the findings of this study are available in the NASA Physical Sciences Informatics (PSI) database, dataset: MICS Launch 1 - Series 5 - C3S (Tricalcium Silicate) - Water to Cement Ratio 2.0. The synthesized 3D datasets in this study are available from the corresponding author upon reasonable request.

## Code availability

The codes for image analysis and microstructure characterization used in this study are available from the corresponding author upon reasonable request.
