# Supplementary material for: Unlocking the potential: analyzing 3D microstructure of small-scale cement samples from space using deep learning
Source: NPJ Microgravity. 2024 Jan 25;10:11. doi: 10.1038/s41526-024-00349-9 (PMC11381549; doi:10.1038/s41526-024-00349-9)
Supplement: Supplementary file 1 — Supplementary Information [file 41526_2024_349_MOESM1_ESM.pdf]

Unlocking the Potential: Analyzing 3D Microstructure of Small-scale Cement Samples from Space using Deep Learning

Vishnu Saseendran[1*], Namiko Yamamoto[1*], Peter J. Collins[2], Aleksandra Radlińska[2], Sara Mueller[3], Enrique M. Jackson[4]

[1] *Department of Aerospace Engineering, The Pennsylvania State University, University Park, 16802 PA, USA.*

[2] *Department of Civil and Environmental Engineering, The Pennsylvania State University, University Park, 16802 PA, USA.*

[3] *Department of Ecosystem Science and Management, The Pennsylvania State University, University Park, 16802 PA, USA.*

[4] *NASA Marshall Space Flight Center, Huntsville, 35808 AL, USA.*

[*] Corresponding author(s). E-mail(s): vms5575@psu.edu; nuy12@psu.edu

Supplementary Notes

1. Micro-CT Imaging

The micro-CT images were acquired using a Zeiss Xradia 620 Versa X-ray Microscope. Scans were performed using parameters that achieved a minimum X-ray count (> 5000) for the unique shape, size, and density of each sample. The scan specifications along with image properties are provided in Supplementary Table 1. The virtual samples were generated using 16-bit cross section images (996 through-thickness slices). For ease of data-handling and post processing, the images were reduced to 8-bit [0 - 255 range], wherein, 0 (black) represents porosity, and 255 (white) represents a solid phase. Additionally, contrast enhancement along with noise-smoothing filter was applied. The threshold bounds to discern each phase was determined from the porosity, CH and C-S-H estimates in Table 1. This post processing was performed by selecting a region of interest (ROI) with the desired edge length. Supplementary Fig. 2(a) shows the entire workflow of micro-CT image processing and volumetric analysis. The phase composition in a micro-CT volume of $1g$ sample (edge length 134.2 μm) is shown in Supplementary Fig. 2(b). Visualization and volumetric analysis were carried out in Dragonfly software [1]. The phase-discerned 3D virtual samples were then evaluated using the low-order probability distribution functions. For instance, the CH phase clustering and connectivity from an extracted ROI in the micro-CT volume can be directly compared to the respective microstructure characteristics generated using the CNN-based model.

2. Low-order Probability Distribution Functions

A brief description of the attributes of individual functions – two-point correlation function, $S_2(r)$, lineal-path function, $L_2(r)$, and two-point cluster function, $C_2(r)$, is provided here using 2D illustration of a Phase i contained within Phase j, see Supplementary Fig. 3 [2].

Two-point Correlation Function, $S_2(r)$*:* For a given set of two random points separated by a distance, r, the two-point correlation function, $S_2(r)$, describes the probability that both points are located within the same phase, and is sensitive to the spatial distribution of a given phase. The limits of $S_2(r)$ is denoted as:

$$S_2(r = 0) = \phi \quad \textit{and} \quad \lim_{r \to \infty} S_2(r) = \phi^2 \tag{1}$$

where, ϕ is the volume fraction of the phase of interest.

Two-point Cluster Function, $C_2(r)$*:* The two-point cluster function, $C_2(r)$, describes the probability that two points can be joined by a nonlinear path that is contained within the same

cluster phase. Thus, compared to $S_2(r)$, $C_2(r)$ is sensitive to the distribution and topological connectivity. The relationship between $S_2(r)$ and $C_2(r)$ can be obtained by decomposing $S_2(r)$ into connected and disconnected parts:

$$S_2(r) = C_2(r) + E_2(r) \tag{2}$$

where, $E_2(r)$ is the probability of finding two points in different clusters. Note that $C_2(r)$ depends only on the magnitude of separation distance, r.

Lineal-path Function, $L_2(r)$: The lineal-path function, $L_2(r)$, determines the probability that a randomly placed line is contained entirely within the same phase (along a specified direction). $L_2(r)$ has been used to describe the characteristics of complex microstructures as it describes the tortuosity of a given phase under consideration [3]. The limits of $L_2(r)$ is denoted as:

$$\lim_{r\to 0} = \phi \quad \mathit{and} \quad \lim_{r\to\infty} L_2(r) = 0 \tag{3}$$

Both porosity and portlandite phases in the reconstructed microstructure of $1g$ and μg samples are compared to their respective spatial distributions and connectivity in the micro-CT virtual samples using the low-order probability functions. A Python script was employed to read-in the image files and evaluate the functions defined in Supplementary Equations 1, 2 and 3.

Supplementary Discussion

Influence of Exemplar on Reconstruction

Two image sizes were extracted from a 1536 x 1024 pixels SEM image in case of both $1g$ and μg samples - 256 x 256 pixels and 512 x 512 pixels, with a resolution 0.54 μm/pixel. As noted before, the CH particles formed on ground samples are relatively smaller in size and are uniformly distributed. Synthesized microstructures along with exemplars are provided in Supplementary Fig. 6 for both sample cases. The synthesized 256^3 voxels μg sample shows the portlandite phase occupying a large portion in the reconstructed volume. In the chosen 256 x 256 pixels exemplar (refer to Supplementary Fig. 5), the occupied area of the portlandite phase is relatively large. The orthogonal view shows porosity occupying interior portion along with the portlandite phase. For reconstructed $1g$ sample volumes, orthogonal views of both 256^3 and 512^3 voxels reveal that the hydrated phases have been very well captured. Hence, for the given resolution, a lower exemplar size may also be utilized for $1g$ sample.

Due to the unique plate-like morphology found in space-returned samples, they are a good candidate to highlight the effect of exemplar size/resolution on 3D reconstruction. Hence, the statistical and quantitative description of the portlandite phase was evaluated for various exemplar sizes using the low-order probability functions, $S_2(r)$, $C_2(r)$, and $L_2(r)$ (see Supplementary Note 3) and was further evaluated against respective micro-CT virtual data. For comparison against micro-CT data, sub-volumes corresponding to size 256^3 and 512^3 voxels were extracted. Supplementary Fig. 7 provides mean plots of probability distribution functions of the portlandite phase for the chosen exemplar sizes of $1g$ and μg sample, respectively.

For the given resolution, in case of the ground-based $1g$ sample, the synthesized volumes using both 256^2 and 512^2 pixels exemplars matched well with the micro-CT virtual volumes. Invariably, in texture synthesis models, the efficacy of the reconstruction is dependent on the inputted target image. For the space-returned μg sample, the chosen 256^2 pixels exemplar (see Supplementary Fig. 6) was randomly selected from a 1536 x 1026 pixels (see Supplementary Fig. 5) BSE image. As shown in Supplementary Fig. 5, the chosen 2D exemplar only partially covers the portlandite phase, which is reflected in the synthesized microstructure, see Supplementary Fig. 6. Therefore, for the given resolution of 0.54 μm/pixel, at least an exemplar size of 512 x 512 pixels is recommended.

Supplementary Table

Supplementary Table 1: *Micro-CT scan specifications and image properties of the μg and $1g$ virtual samples.*

	μg Sample	$1g$ Sample
System	Zeiss Xradia 620 Versa	
Voltage [kV]	50	70
Power [W]	4.5	8.5
Exposure Time [s]	4	2
Source to RA Distance [mm]	12.02	16.22
Detector to RA distance [mm]	29.31	32.14
Optical Magnification	4x	
Binning	2	
Filter	LE3†	LE5†
Image Properties		
Pixel size [μm]	2.0 (μg), 2.2 ($1g$)	
Bits per pixel	16	
Width	989 pixels	
Height	1013 pixels	
Depth	997 pixels	
Voxels	999,861,390	

† `LE3` and `LE5` correspond to the Zeiss proprietary low energy filter 3 and 5, respectively.

Supplementary Figures

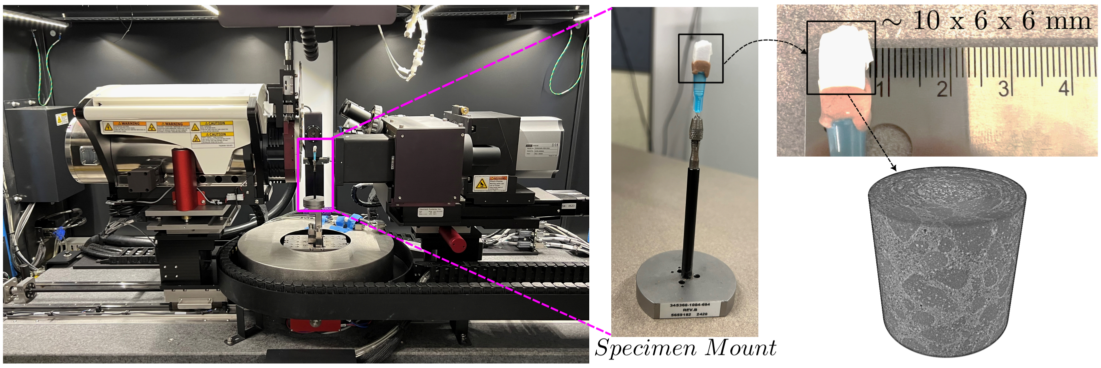

(a) Micro-CT scan setup

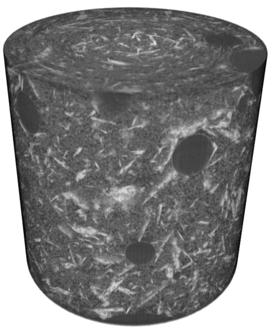

(b) μg virtual sample

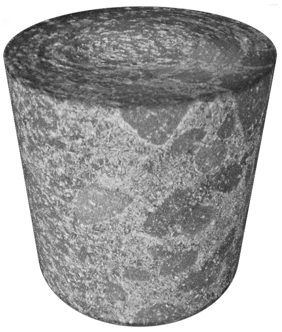

(c) $1g$ virtual sample

Supplementary Figure 1: *Micro-CT scan setup: (a) the hydrated C_3S samples were mounted atop a specimen holder. The acquired pixel resolution is 2.0 and 2.2 μm for (b) μg and (c) $1g$ samples, respectively.*

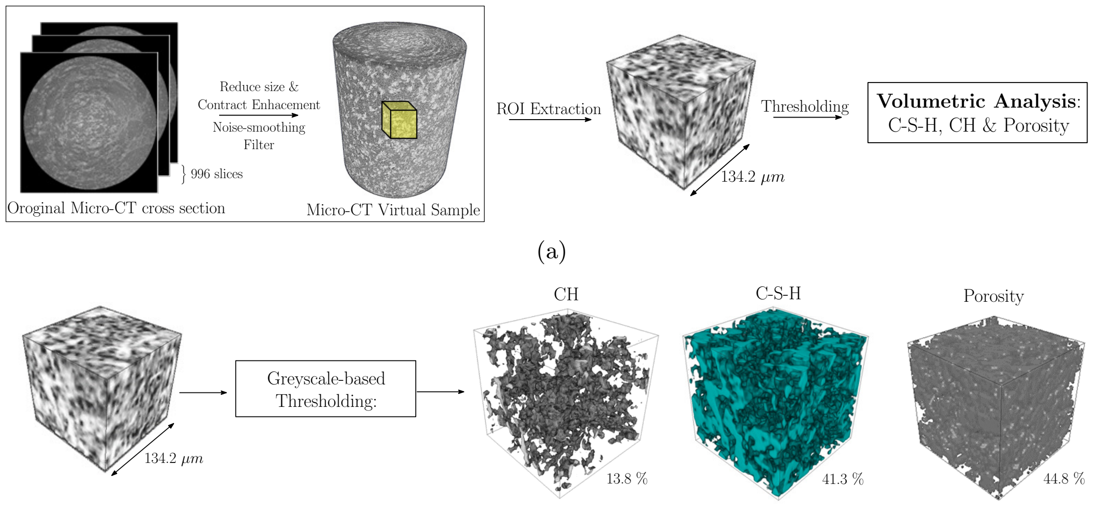

(b)

Supplementary Figure 2: *(a) Workflow of micro-CT image processing, ROI extraction and analysis procedure. Extracted 3D virtual sample of edge length 134 μm (1g sample) is shown here., (b) Greyscale histogram-based thresholding used to determine each phase in a micro-CT volume with edge length 134 μm (1g sample). Individual phase composition is noted alongside each sub-volume.*

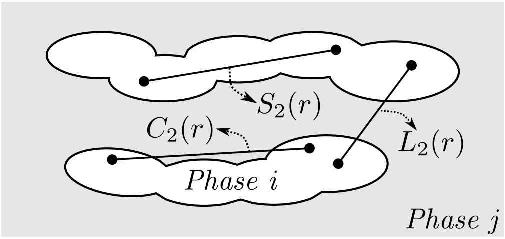

Supplementary Figure 3: *Schematic illustration of low-order probability functions – $S_2(r)$, $L_2(r)$ and $C_2(r)$, that defines the spatial correlation of inclusions of Phase i within Phase j [2].*

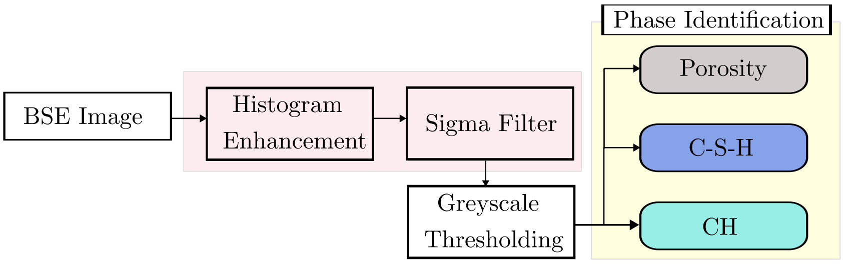

Supplementary Figure 4: *Workflow of greyscale histohram-based BSE image (1536* x 1024 pixels) segmentation for phase identification in hydrated 1g and μg C_3S samples.

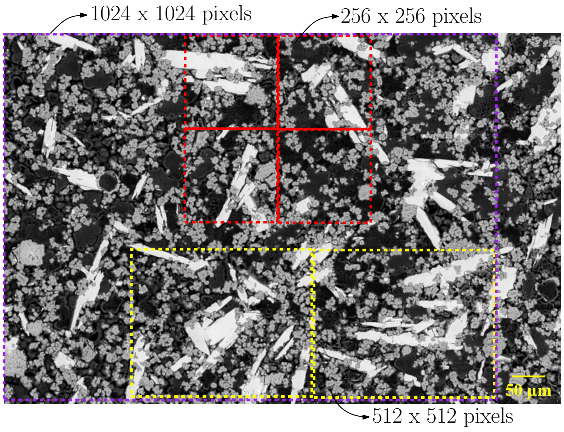

Supplementary Figure 5: *Selection of 2D exemplars from an 1536 x 1026 pixels BSE image of the space-returned sample with a resolution 0.54 µm/pixel.*

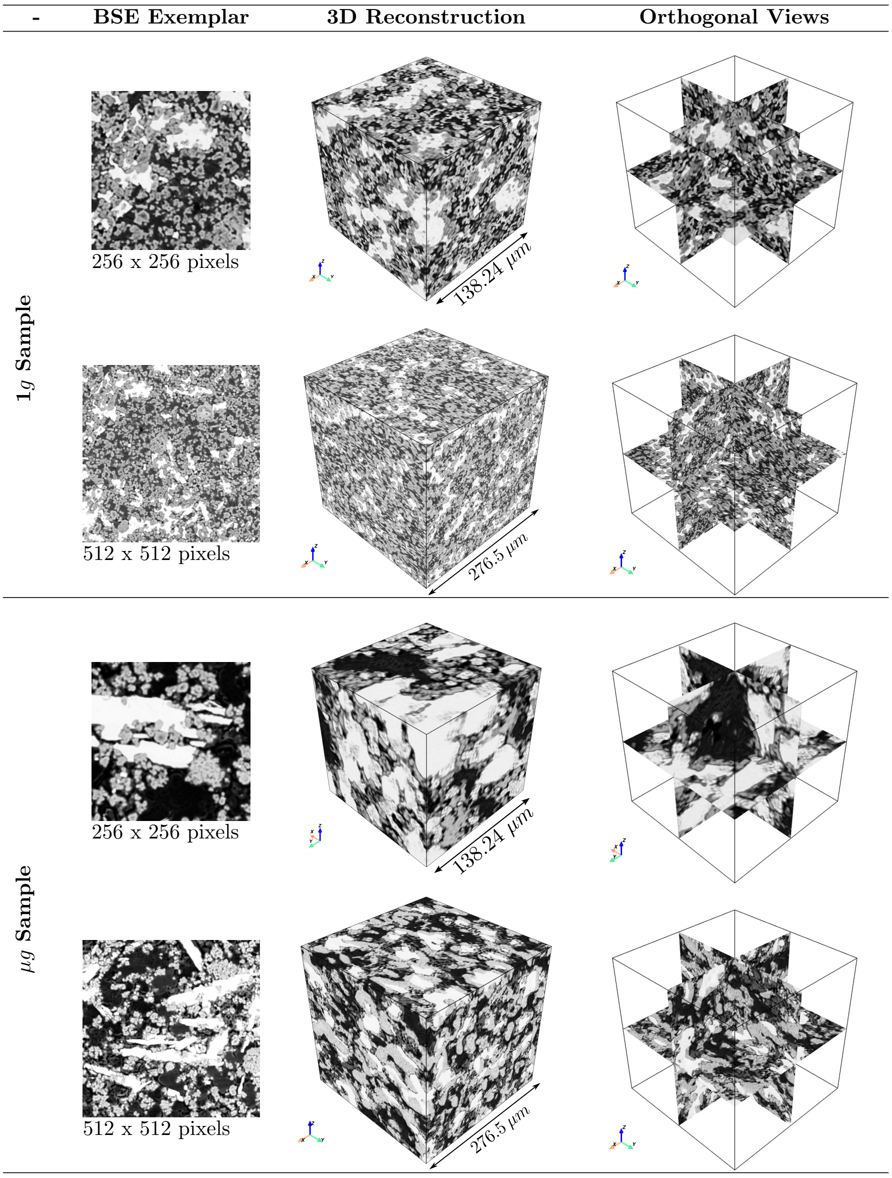

Supplementary Figure 6: *Effect of exemplar size: qualitative comparison of reconstructed 3D microstructure and their respective orthogonal views along with the 2D exemplars for both 1g and µg samples. Exemplar sizes considered - 256 x 256 pixels and 512 x 512 pixels (resolution 0.54 µm).*

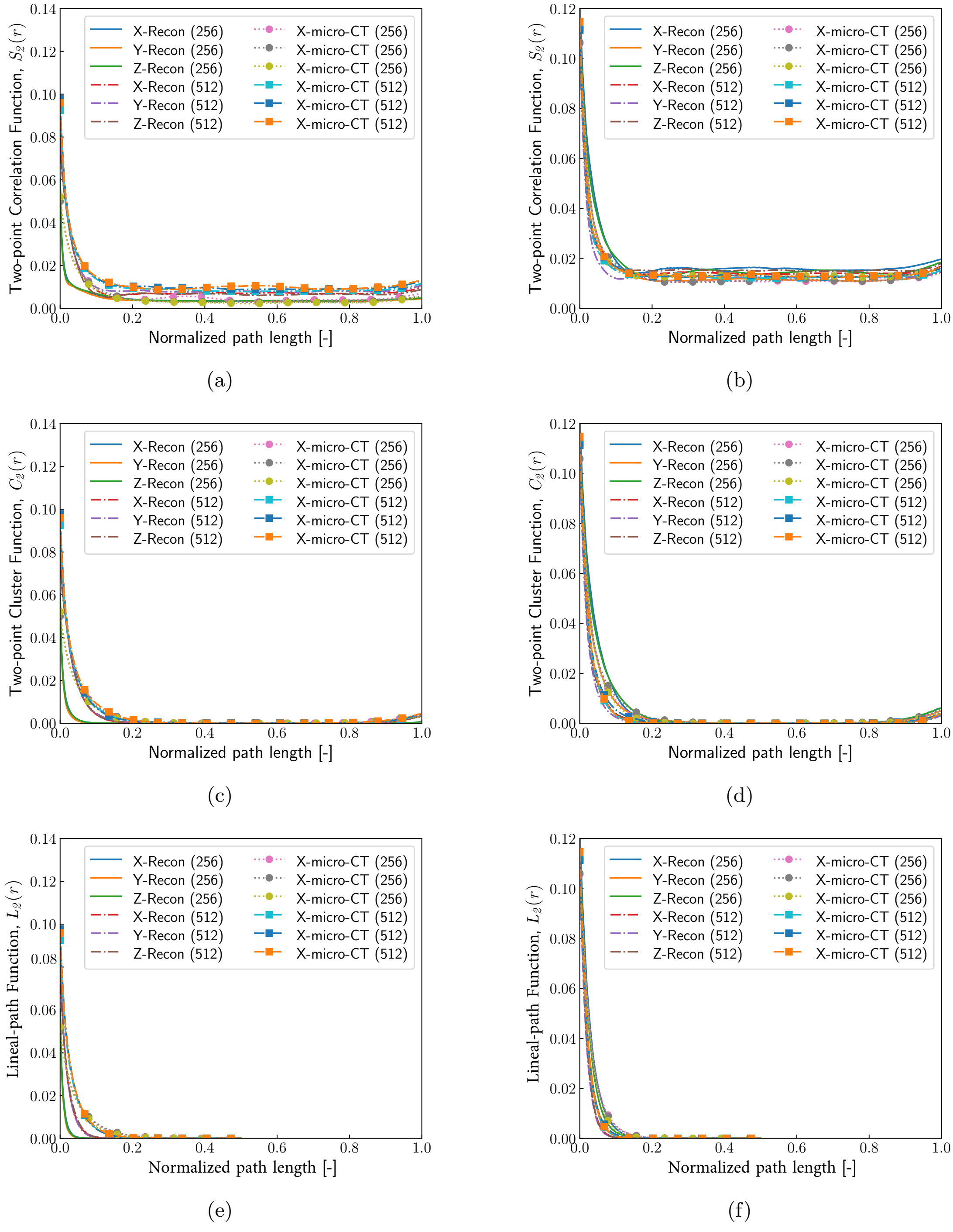

Supplementary Figure 7: *Impact of exemplar size in microstructure reconstruction - Quantitative comparison of portlandite phase using statistical descriptors: two-point correlation function, $S_2(r)$ (a) µg sample, (b) 1g sample; two-point cluster function, $C_2(r)$ (c) µg sample, (d) 1g sample and lineal-path function, $L_2(r)$ (e) µg sample, (f) 1g sample. For 2D exemplars, see Supplementary Fig. 6.*

Supplementary References

[1] Object Research Systems (ORS) Inc. Dragonfly 2022.1 [computer software]. URL http://www.theobjects.com/dragonfly.

[2] Torquato, S. & Haslach Jr, H. Random heterogeneous materials: Microstructure and Macroscopic properties. *Appl. Mech. Rev.* **55**, B62–B63 (2002).

[3] Lu, B. & Torquato, S. Lineal-path function for random heterogeneous materials. *Phys. Rev. A* **45**, 922 (1992).
